# Prognostic significance of laterality in renal cell carcinoma: A population‐based study from the surveillance, epidemiology, and end results (SEER) database

**DOI:** 10.1002/cam4.2484

**Published:** 2019-08-12

**Authors:** Shengjie Guo, Kai Yao, Xiaobo He, Shulin Wu, Yunlin Ye, Junxing Chen, Chin‐Lee Wu

**Affiliations:** ^1^ Department of Urology, Sun Yat‐Sen University Cancer Center State Key Laboratory of Oncology in South China Collaborative Innovation Center for Cancer Medicine Guangzhou China; ^2^ Department of Urology and Pathology Massachusetts General Hospital Harvard Medical School Boston MA USA; ^3^ Glickman Urological and Kidney Institute, Cleveland Clinic Cleveland OH USA; ^4^ Department of Urology The First Affiliated Hospital Sun Yat‐Sen University Guangzhou China

**Keywords:** laterality, nephrectomy, prognosis, renal cell carcinoma, SEER

## Abstract

**Background:**

Various prognostic characteristics have been established in the renal cell carcinoma (RCC). However, the impact of tumor laterality is unknown. The objective of the current study was to explore the predictive and prognostic impact of tumor laterality of RCC after surgery.

**Methods:**

This investigation was a population‐based retrospective cohort study of patients with RCC from the surveillance, epidemiology, and end results (SEER) database in the USA. All patients received surgical treatment between January 2010 and December 2014. Cancer‐specific survival (CSS) measured from the time of surgery.

**Results:**

This study identified 41 138 surgically treated RCC patients: Of these patients, 50.6% had right‐sided RCC, 59.5% were younger than 65 years of age, 63.8% were male, and 81.0% were Caucasian. The stage distribution was 67.0% (I), 9.5% (II), 17.1% (III), and 6.4% (IV). Patients with right‐sided RCC were more likely to have favorable clinicopathological features compared with patients with left‐sided RCC. In adjusted analyses, patients with right‐sided RCC showed significantly better CSS than those with left‐sided RCC within different subgroups including tumor size ≥10 cm (*P* = .004), age <65 years (*P* = .002), male gender (*P* = .001), Caucasian race (*P* = .001), clear cell carcinoma type (*P* = .024), and radical nephrectomy (*P* = 0.008). Moreover, in the subgroup of tumor size ≥10 cm, right‐sided cancer was an independent predictor of CSS (*P* = .022).

**Conclusion:**

Right‐sided RCC is associated with more early‐stage, low‐grade disease and shows better CSS than left‐sided RCC. Moreover, laterality remained as an independent prognostic factor for cancer‐specific survival in subgroup of tumor size ≥10 cm RCC.

## Graphical abstract

Renal cell laterality could be a prognostic factor for cancer‐specific survival in subgroup of tumor size ≥10 cm RCC. Right‐sided RCC is associated with more early‐stage, low‐grade disease and shows better CSS than left‐sided RCC.

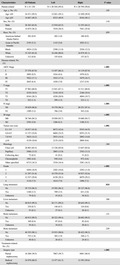

## 1 BACKGROUND

Renal cell carcinoma (RCC), a heterogeneous type of cancer originating from the nephron, accounts for approximately 3.9% of new carcinomas, with an increasing incidence in the past two decades because of the wide application of ultrasound and computed tomography.^1^, ^2^, ^3^ According to the United States Cancer Statistics, an estimated 63 990 new kidney cancer cases will occur, and 14 400 patients died from kidney cancer in 2017.^4^ In‐depth studies in the field of RCC preferably describe renal cancer using various characteristics, such as age, Karnofsky Performance Scale (KPS), histologic grade, tumor size, disease stage, and histological subtype, given the prognostic value of these factors in RCC survival.^5^, ^6^ However, tumor location is not regarded as a universally acknowledged prognostic factor in cancer‐specific survival unlike the above indices. How the primary tumor location influences disease outcome remains controversial. Anatomically, the kidneys are located retroperitoneally in the posterior abdominal wall and are found between the transverse processes of T12 and L3. Furthermore, the right kidney is most commonly found to be lower in the abdomen than in the left kidney.^7^ Clinically, previous studies have suggested that the proportion of tumors occurring showed an equal or near‐equal distribution between the right and left kidneys.^8^, ^9^ A previous study^9^ showed that patients with right‐sided tumors had a prolonged survival compared with those with left‐sided tumors, whereas another study^10^ showed an improved 5‐year survival rate for patients with left‐sided tumors.

To determine whether primary tumor laterality independently contributes to RCC prognosis, we evaluated the impact of laterality on cancer‐specific survival (CSS) using the surveillance, epidemiology, and end results (SEER) database.

## 2 METHODS

### 2.1 Study design and study population

**Figure 1:**
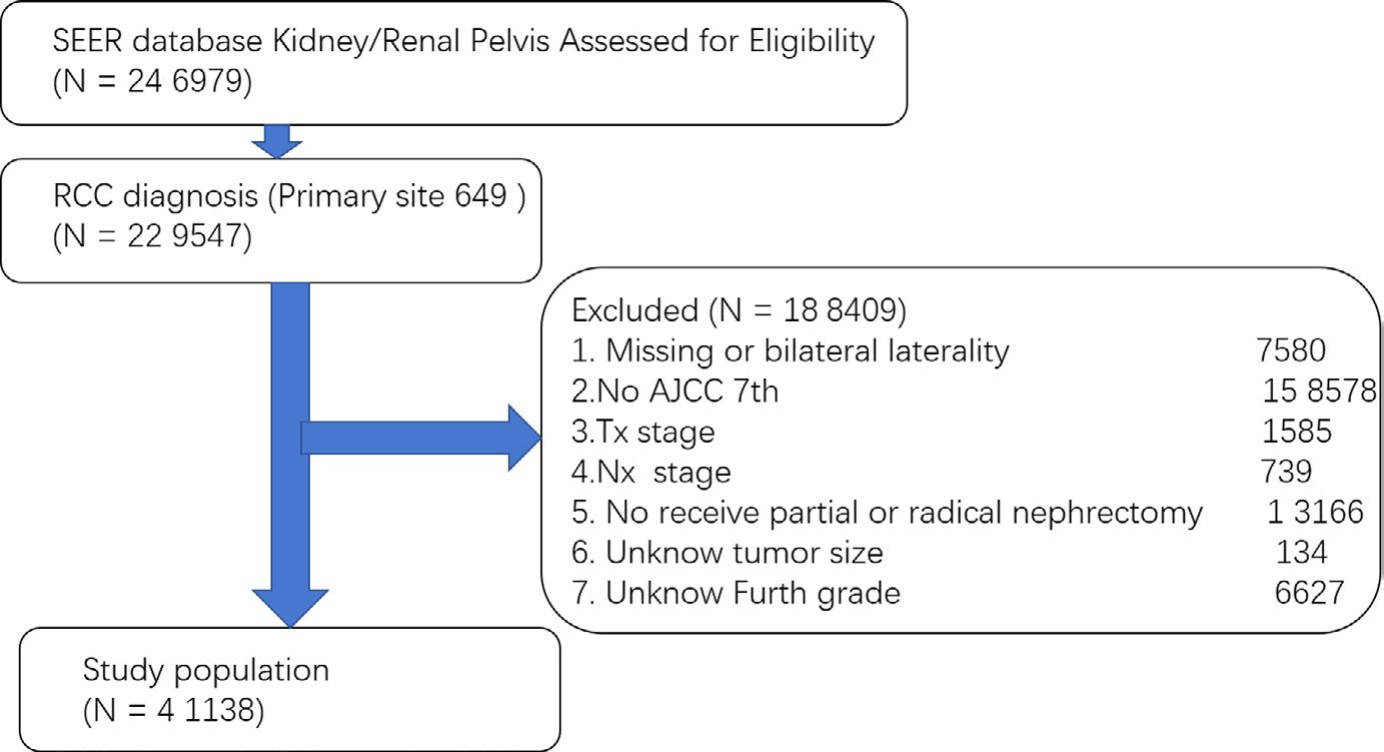
Cohort selection. Of 246 979 patients with RCC identified in SEER database 205,841 were excluded for reasons shown

We collected renal cancer records from the population‐based SEER program of the US National Cancer Institute, which includes cancer registries covering 28% of the US population.^11^ We identified a source population of 246 979 patients in the SEER registry who were diagnosed with kidney cancer and 229 547 patients who were diagnosed with RCC (primary site: 649). The specific exclusion criteria were as follows: missing or bilateral laterality of tumors, no definite American Joint Committee on Cancer (AJCC) stage (7th edition), obtaining Tx stage or Nx stage derived from the 7th AJCC stage group, untreated with partial nephrectomy (Code 30) or radical nephrectomy (Code 40 and 50), unknown tumor size and unknown grade. After applying the exclusion criteria, 41 138 patients were included in the analysis (Figure 1).

### 2.2 Measures and outcomes

The primary outcome was CSS. The survival time was calculated from the date of RCC diagnosis to the date of death or date that the patient was last known to be alive, or the last follow‐up, whichever occurred first. Covariates of interest included the age at diagnosis (age <65 years or age ≥65 years), gender, race (American Indian/Alaska Native, Asian or Pacific Islander, Black, White, or Unknown), AJCC stage (I‐IV), T stage (T1‐4), N stage (N0 or N1), M stage (M0 or M1), tumor size (0.1 to 3.9, 4.0 to 6.9, 7.0 to 9.9, or 10 cm or greater), histological variant (clear cell, papillary, collecting duct, chromophobe, or other), grade (well‐differentiated, moderately differentiated, poorly differentiated or undifferentiated), laterality (left or right), and surgical type (partial nephrectomy or radical nephrectomy).

### 2.3 Statistical analysis

Chi‐squared test was used to compare the clinicopathological characteristics between primary tumor laterality. Adjusted hazard ratio (HR) along with 95% confidence interval (CI) were calculated using the Cox proportional hazards regression model. All statistical analyses mentioned above were performed using the Statistic Package for Social Science (SPSS), version 24.0 (SPSS Inc). Two‐sided *P* values less than .05 were considered statistically significant.

## 3 RESULTS

### 3.1 Patient characteristics

The characteristics of the patients are summarized in Table 1. Among the 41,138 patients, 50.6% had right‐sided RCC, 59.5% were younger than 65 years of age, 63.8% were male, and 81.0% were Caucasian race. Most patients were diagnosed with stage I disease (67.0%), followed by stage III disease (17.1%), stage II disease (9.5%) and stage IV disease (6.4%). Radical nephrectomy was performed in 60.5% of patients.

**Table 1:** Characteristics of 41 138 patients with renal cell carcinoma in SEER between 2010 and 2014

Characteristics	All Patients	Left	Right	*P* value
Patient‐related	N = 41 138	N = 20 344 (49.4)	N = 20 794 (50.6)	
Age, y, No. (%)				.105
Age <65	24 471 (59.5)	12 021 (59.1)	12 450 (59.9)	
Age ≥65	16 667 (40.5)	8323 (40.9)	8344 (40.1)	
Sex, No. (%)				.110
Male	26 263 (63.8)	12 910 (63.5)	13 353 (64.2)	
Female	14 875 (36.2)	7434 (36.5)	7441 (35.8)	
Race, No. (%)				.059
American Indian/Alaska Native	382 (0.9)	202 (1.0)	180 (0.9)	
Asian or Pacific Islander	2195 (5.3)	1143 (5.6)	1052 (5.1)	
Black	4924 (12.0)	2398 (11.8)	2526 (12.2)	
White	33 325 (81.0)	16 446 (80.8)	16 879 (81.1)	
Unknown	312 (0.8)	155 (0.8)	157 (0.7)	
Disease‐related, No. (%)				
AJCC Stage				<.001*
I	27 576 (67.0)	13 457 (66.2)	14 119 (67.9)	
II	3895 (9.5)	1916 (9.4)	1979 (9.5)	
III	7022 (17.1)	3543 (17.4)	3479 (16.7)	
IV	2645 (6.4)	1428 (7.0)	1217 (5.9)	
T stage				<.001*
T1	27 962 (68.0)	13 651 (67.1)	14 311 (68.8)	
T2	4310 (10.4)	2144 (10.5)	2166 (10.4)	
T3	8344 (20.3)	4250 (20.9)	4094 (19.7)	
T4	522 (1.3)	299 (1.5)	223 (1.1)	
N stage				<.001*
N0	39 829 (96.8)	19 578 (96.2)	20 251 (97.4)	
N1	1309 (3.2)	766 (3.8)	543 (2.6)	
M stage				<.001*
M0	38 746 (94.2)	19 058 (93.7)	19 688 (94.7)	
M1	2392 (5.8)	1286 (6.3)	1106 (5.3)	
Tumor size (cm)				<.001*
0.1‐3.9	18 017 (43.8)	8672 (42.6)	9345 (44.9)	
4.0‐6.9	13 157 (32.0)	6604 (32.5)	6553 (31.5)	
7.0‐9.9	5825 (14.2)	2933 (14.4)	2892 (14.0)	
≥10	4139 (10.0)	2135 (10.5)	2004 (9.6)	
Histology				.368
Clear cell	26 883 (65.4)	13 236 (65.0)	13 647 (65.6)	
Papillary	5566 (13.5)	2803 (13.8)	2763 (13.3)	
Collecting duct	72 (0.2)	41 (0.2)	31 (0.2)	
Chromophobe	1902 (4.6)	930 (4.6)	972 (4.6)	
Other specified	6715 (16.3)	3334 (16.4)	3381 (16.3)	
Grade				<.001*
1	4494 (10.9)	2202 (10.8)	2292 (11.0)	
2	21 297 (51.8)	10 370 (51.0)	10 927 (52.6)	
3	12 237 (29.8)	6158 (30.3)	6079 (29.2)	
4	3110 (7.5)	1614 (7.9)	1496 (7.2)	
Lung metastasis				.024*
No	39 719 (96.5)	19 592 (96.3)	20 127 (96.8)	
Yes	1340 (3.3)	709 (3.5)	631 (3.0)	
Unknown	79 (0.2)	43 (0.2)	36 (0.2)	
Liver metastasis				.306
No	40 815 (99.2)	20 171 (99.2)	20 644 (99.3)	
Yes	270 (0.7)	146 (0.7)	124 (0.6)	
Unknown	53 (0.1)	27 (0.1)	26 (0.1)	
Brain metastasis				.121
No	40 915 (99.5)	20 223 (99.4)	20 692 (99.5)	
Yes	169 (0.4)	87 (0.4)	82 (0.4)	
Unknown	54 (0.1)	34 (0.2)	20 (0.1)	
Bone metastasis				.229
No	40 355 (98.1)	19 933 (98.0)	20 422 (98.2)	
Yes	733 (1.8)	385 (1.9)	348 (1.7)	
Unknown	50 (0.1)	26 (0.1)	24 (0.1)	
Treatment‐related, No. (%)				
Surgery type				<.001*
Partial nephrectomy	16 268 (39.5)	7867 (38.7)	8401 (40.4)	
Radical nephrectomy	24 870 (60.5)	12 477 (61.3)	12 393 (59.6)	

Abbreviation: AJCC, American Joint Committee on Cancer.

* It was statistically significant.

Patients with right‐sided RCC were more likely to have early‐stage disease (I and II) (77.4% vs 75.6%; *P* < .001), a tumor size <4 cm (44.9% vs 42.6%; *P* < .001), negative lymph node metastasis (97.4% vs 96.2%; *P* < .001), negative organ metastasis (94.7% vs 93.7%; *P* < .001) and well‐ or moderately differentiated tumors (63.6% vs 61.8%; *P* < .001) than patients with left‐sided RCC. Moreover, a higher proportion of right‐sided RCC patients received partial nephrectomy (61.3% vs 59.6%; *P* < .001).

### 3.2 Factors associated with survival

Based on univariate analysis, right‐sided RCC shows better survival than left‐ sided RCC (HR: 0.86; 95% CI: 0.79‐0.94; *P* = .001). However, on multivariate analysis, laterality had no significant effect on RCC for CSS (HR: 0.96; 95% CI: 0.88‐1.04; *P* = .354) (Table 2).

**Table 2:** Factors associated with cancer‐specific survival among 41 138 patients with renal cell carcinoma in SEER between 2010 and 2014

Covariate	Univariate analysis		*P* value	Multivariate analysis		*P* value
	HR	95% CI		HR	95% CI	
Age, y	1.02	1.01‐1.02	<.001*	1.01	1.00‐1.01	<.001*
Sex						
Female	Ref			Ref		
Male	1.28	1.16‐1.40	<.001*	0.98	0.89‐1.08	.787
Race						
American Indian/Alaska Native	Ref			Ref		
Asian or Pacific Islander	1.89	1.09‐3.27	.023*	1.83	1.05‐3.18	.031*
Black	1.24	0.72‐2.14	.430	1.85	1.07‐3.19	.025*
White	1.53	0.90‐2.58	.114	1.71	1.01‐2.90	.045*
Unknown	0.12	0.02‐0.90	.039*	0.18	0.02‐1.42	.105
AJCC Stage						
I	Ref			Ref		
II	3.40	2.75‐4.19	<.001*	1.19	0.93‐1.52	.146
III	10.1	8.73‐11.60	<.001*	3.49	2.94‐4.14	<.001*
IV	63.9	55.96‐73.16	<.001*	15.41	12.92‐18.38	<.001*
T stage						
T1	Ref			Ref		
T2	5.01	4.28‐5.86	<.001*	—	—	—
T3	13.0	11.59‐14.64	<.001*	—	—	—
T4	55.5	46.88‐65.67	<.001*	—	—	—
N stage						
N0	Ref			Ref		
N1	16.5	14.93‐18.22	<.001*	—	—	—
M stage						
M0	Ref			Ref		
M1	21.7	19.90‐23.70	<.001*	—	—	—
Tumor size (cm)						
0.1‐3.9	Ref			Ref		
4.0‐6.9	4.52	3.78‐5.41	<.001*	1.83	1.51‐2.22	<.001*
7.0‐9.9	13.2	11.04‐15.69	<.001*	2.37	1.91‐2.94	<.001*
≥10	28.3	23.88‐33.58	<.001*	3.01	2.43‐3.72	<.001*
Histology						
Clear cell	Ref			Ref		
Papillary	0.74	0.63‐0.86	.001*	1.45	1.24‐1.71	<.001*
Collecting duct	9.84	6.46‐15.01	<.001*	3.00	1.96‐4.60	<.001*
Chromophobe	0.31	0.21‐0.44	<.001*	0.47	0.32‐0.68	<.001*
Other specified	2.12	1.92‐2.33	<.001*	1.58	1.43‐1.75	<.001*
Grade						
1	Ref			Ref		
2	2.02	1.46‐2.78	<.001*	1.35	0.97‐1.86	.068
3	8.70	6.36‐11.90	<.001*	2.73	1.98‐3.76	<.001*
4	40.8	29.78‐55.82	<.001*	5.00	3.62‐6.93	<.001*
Surgery type						
Partial nephrectomy	Ref			Ref		
Radical Nephrectomy	9.75	8.26‐11.50	<.001*	1.91	1.58‐2.03	<.001*
Laterality						
Left	Ref			Ref		
Right	0.86	0.79‐0.94	.001*	0.96	0.88‐1.04	.354

Abbreviations: AJCC, American Joint Committee on Cancer; HR, hazard ratio.

* It was statistically significant.

In the univariate subgroup analysis, CSS was better in right‐sided RCC patients with age <65 years at diagnosis (HR: 0.84; 95% CI: 0.75‐0.94; *P* = .002), male gender (HR: 0.85; 95% CI: 0.77‐0.94; *P* = .001), Caucasian race (HR: 0.86; 95% CI: 0.79‐0.94; *P* = .001), tumor size ≥10 cm (HR: 0.83; 95% CI: 0.72‐0.94; *P* = .004), clear cell carcinoma type (HR: 0.88; 95% CI: 0.79‐0.98; *P* = .024), grade 3 differentiated (HR: 0.85; 95% CI: 0.74‐0.96; *P* = .004), no lung metastasis (HR: 0.89; 95% CI: 0.81‐0.99; *P* = .024), no bone metastasis (HR: 0.86, 95% CI: 0.79‐0.94; *P* = .001), and radical nephrectomy (HR: 0.89; 95% CI: 0.82‐0.97; *P* = .008) than patients with left‐sided RCC. However, no association was found between laterality and CSS at any stage of RCC (Table 3). Additionally, we analyzed the different subgroups of age, race, tumor size, pathology, grade, and surgery by multivariate analysis for CSS. There was no statistical significance for different subgroups to identify that laterality was an independent factor (Table S1).

**Table 3:** Univariate analysis of laterality in different subgroups of patients with renal cell carcinoma in SEER between 2010 and 2014 for cancer‐specific survival

Covariate	Univariate analysis (Left vs Right)		
	HR	95% CI	*P* value
Age, y			
Age <65	0.84	0.75‐0.94	.002*
Age ≥65	0.91	0.81‐1.03	.135
Sex, No. (%)			
Male	0.85	0.77‐0.94	.001*
Female	0.91	0.79‐1.06	.217
Race, No. (%)			
American Indian/Alaska Native	0.76	0.26‐2.19	.610
Asian or Pacific Islander	0.98	0.71‐1.36	.922
Black	0.90	0.69‐1.16	.403
White	0.86	0.79‐0.94	.001*
Unknown	2.07	0.19‐22.85	.552
AJCC stage			
I	0.96	0.77‐1.20	.729
II	1.11	0.80‐1.53	.522
III	0.95	0.82‐1.10	.457
IV	0.92	0.82‐1.04	.186
T stage			
T1	0.95	0.78‐1.15	.580
T2	0.94	0.75‐1.18	.576
T3	0.90	0.81‐1.01	.063
T4	0.91	0.70‐1.19	.497
N stage			
N0	0.94	0.86‐1.03	.206
N1	0.92	0.78‐1.08	.313
M stage			
M0	0.94	0.84‐1.05	.244
M1	0.91		.130
Tumor size (cm)			
0.1‐3.9	0.97	0.72‐1.31	.850
4.0‐6.9	0.97	0.82‐1.15	.758
7.0‐9.9	0.95	0.82‐1.11	.530
≥10	0.83	0.72‐0.94	.004*
Histology			
Clear cell	0.88	0.79‐0.98	.024*
Papillary	0.86	0.66‐1.13	.281
Collecting duct	1.50	0.70‐3.22	.297
Chromophobe	0.52	0.25‐1.10	.086
Other specified	0.88	0.76‐1.02	.085
Grade			
1	1.05	0.60‐1.83	.855
2	0.85	0.70‐1.03	.104
3	0.85	0.74‐0.96	.010*
4	1.00	0.88‐1.15	.966
Lung metastasis			
No	0.89	0.81‐0.99	.024*
Yes	0.87	0.75‐1.02	.089
Unknown	2.10	0.91‐4.88	.083
Liver metastasis			
No	0.88	0.81‐0.96	.004*
Yes	0.76	0.55‐1.05	.096
Unknown	1.55	0.62‐3.86	.346
Brain metastasis			
No	0.87	0.80‐0.95	.001*
Yes	0.79	0.52‐1.19	.261
Unknown	0.97	0.35‐2.67	.948
Bone metastasis			
No	0.86	0.79‐0.94	.001*
Yes	1.02	0.83‐1.27	.835
Unknown	0.85	0.31‐2.36	.761
Surgery type			
Partial nephrectomy	0.87	0.63‐1.19	.375
Radical nephrectomy	0.89	0.82‐0.97	.008*

Abbreviations: AJCC, American Joint Committee on Cancer; HR, hazard ratio.

* It was statistically significant.

We undertook further analysis within the different tumor size subgroups to identify prognostic factors that may differ between left‐sided and right‐sided RCC. Multivariate Cox proportional hazards regression model analysis showed no association between laterality and CSS in the subgroups of tumor size <4 cm, 4 cm ≤ tumor size <7 cm, and 7 cm ≤ tumor size <10 cm (Table S2, Table S3 and Table S4). However, in the subgroup of tumor size ≥10 cm, right‐sided cancer was an independent predictor of CSS (HR: 0.85; 95% CI: 0.75‐0.97; *P* = 0.022), as well as AJCC stage, histology, and grade (Table 4).

**Table 4:** Multivariate analysis in group of patients with renal cell carcinoma (tumor size ≥10 cm) in SEER between 2010 and 2014 for cancer‐specific survival

Covariate	Multivariate analysis		
	HR	95% CI	*P* value
Age, y			
Age <65 (N = 2589)	Ref		
Age ≥65 (N = 1550)	1.08	0.94‐1.23	.267
Sex, No. (%)			
Male (N = 2863)	Ref		
Female (N = 1276)	1.05	0.91‐1.21	.447
AJCC Stage			
II (N = 1397)	Ref		
III (N = 1579)	3.65	2.76‐4.83	<.001*
IV (N = 1163)	11.1	8.44‐14.6	<.001*
Histology			
Clear cell (N = 2517)	Ref		
Papillary (N = 435)	1.50	1.16‐1.93	.002*
Collecting duct (N = 12)	2.99	1.33‐6.72	.008*
Chromophobe (N = 269)	0.54	0.33‐0.90	.018*
Other specified (N = 906)	1.64	1.42‐1.90	<.001*
Grade			
1 (N = 129)	Ref		
2 (N = 1195)	5.19	1.28‐21.0	.021*
3 (N = 1764)	9.61	2.39‐38.6	.001*
4 (N = 1051)	15.1	3.74‐60.6	<.001*
Surgery type			
Partial nephrectomy (N = 152)	Ref		
Radical nephrectomy (N = 3987)	1.04	0.66‐1.63	.857
Laterality			
Left (N = 2589)	Ref		
Right (N = 1550)	0.85	0.75‐0.97	.022*

Abbreviations: AJCC, American Joint Committee on Cancer; HR, hazard ratio.

* It was statistically significant.

## 4 DISCUSSION

In this population‐based cohort study, we aimed to explore whether laterality was associated with survival among patients with surgical treatment for RCC. Some important findings were revealed in this study. First, right‐sided RCC is more likely to have an early tumor stage and lower tumor grade than left‐sided RCC. Second, the tumor size <4 cm and partial nephrectomy treatment are more likely in right‐sided RCC. Third, the right‐sided RCC has better CSS than left‐sided disease in subgroups which with a tumor size ≥10 cm, age <65 years, male gender, Caucasian race, clear cell carcinoma type, grade 3 differentiation, no lung metastasis, no bone metastasis, and radical nephrectomy. Moreover, laterality was an independent prognostic factor of survival in the tumor size ≥10 cm subgroup.

Due to the difference in the embryonic sources, anatomy, blood supply, lymphatic drainage, and relationship to the surrounding organs, the incidence of cancer may vary with laterality. It has been shown that the breast cancer incidence is higher on the left side and that of testicular cancer is higher on the right side.^12^ The incidence of RCC is similar with right‐sided and left‐sided RCC, while the female gender is associated with a higher incidence in the early stage in right‐sided than left‐sided RCC.^1^ In this study, the incidence of right‐sided RCC was slightly higher than that of left‐sided RCC (50.6% vs 49.4%, respectively). In the tumor stage subgroups, Katkoori et al^13^ showed that right‐sided RCC was associated with a higher ratio of level III inferior vena cava (IVC) tumor thrombus (T3b) patients than with left‐sided RCC. Other reports have shown incidence rates of right‐sided IVC tumor thrombus of 53.5%, 64% and 81%.^14^, ^15^, ^16^ Conversely, fewer patients had stage T3b disease on the right side than on the left side in this study. A similar trend was observed for stage T4, lymph node and organ metastasis RCC patients. Therefore, right‐sided RCC showed a lower advanced stage than left‐sided RCC in this study.

Our findings are consistent with those of Deluhant et al's study. In which they found that disease laterality was associated with survival in a cohort of 1308 patients.^9^ The primary outcome in their study showed that right‐sided RCC was associated with better survival than left‐sided RCC, in which the 5‐year survival for right‐sided RCC was 47.6% and that for left‐sided RCC was 39.6% (*P* = .034). In subgroup analysis, they found that tumor laterality was stage‐dependent, with significantly increased survival for right‐sided tumors in Stage II RCC only (*P* = .007). It was explained that right‐sided tumors are more localized, and surgically treated tumors suggest that radical nephrectomy is more likely to result in tumor clearance for right‐sided RCC than for tumors on the left side.^9^ These findings were similar to ours, in which more patients with right‐sided tumors had stage I/II disease, negative lymph node or organ metastasis, tumor size <4 cm, tumor grade 1/2 and partial nephrectomy than those with left‐sided tumors. Therefore, the CSS was better in right‐sided tumors than in left‐sided RCC. Moreover, laterality was an independent prognostic factor adjusted for the tumor stage and histology in the subgroup of tumor size ≥10 cm. Stage II in the above report was defined as regional or lymph node involvement, which is similar to stage III in the 7th AJCC edition. Although we evaluated stage III subgroups in our study, no significant difference was found in CSS regarding laterality. The major limitation of their study was that not all the patients received surgical treatment and the number of patients enrolled was relatively small. Moreover, the RCC survival rate of their report was much lower than in our study, in which the 1‐year and 5‐survival rates were 60.5% and 42.4%, respectively; in our study, the 1‐year and 48‐month survival rates were 94.5% and 89.5%, respectively. The improved survival may be due to the early diagnosis of RCC, surgical technique progression and use of adjusted treatment of target therapy for metastatic disease since 2005. Therefore, the patient enrollment dates of this study between 2010 and 2014 are more reliable for the analysis of laterality with the survival of RCC.

However, the report by Katkoor et al^13^ demonstrated that survival did not differ with laterality of the RCC patients. It was notable that their study only included inferior vena cava stage, a small number of patients (87 cases), and data from a single center. Consistent with our results, T3 stage showed a similar CSS regarding laterality. There was a trend of a decreased survival rate according to the increased tumor size, consistent with our study.^17^ In the tumor size ≥10 cm subgroup, more early stage II disease, equal stage III disease, and less stage IV disease, lymph node metastasis or organ metastasis were observed in right‐sided RCC than in left‐sided RCC.

The mechanism by which the patients with right‐sided RCC showed better survival remains unclear, especially those in the ≥10 cm tumor size subgroup. In this study, the cause may be the lower tumor grade (1/2), earlier tumor stage, and smaller tumor size (<4 cm) for the right side, indicating less invasive and more localized RCC. Moreover, more vascular collateral circulation in the left renal vein, which collects the lumbar, gonadal and adrenal veins, may induce more metastasis. Therefore, left‐sided tumors might have more lung metastasis than right‐sided tumors. At the same time, the lymph positive rate was lower in right‐sided tumors, and the survival of N+ patients was much lower than those without lymph node metastasis. Therefore, lower tumor grade, smaller tumor size, earlier disease stage, and less lymph node, and organ metastasis may contribute to better survival of right‐sided RCC than left‐sided disease.

Several prognostic factors have been identified for RCC, among which tumor node metastasis classification remains the most important.^18^ This trend contrasts colon cancer, which has different embryological origins between right‐ and left‐sided disease. At the same time, the microsatellite instability (MSI) and BRAF mutations are both known to be closely related to right‐sided colon cancer and its poor prognosis. However, no clinically useful “biomarker” has predicted the prognosis of RCC patients until now.

It is important to highlight that this study evaluated CSS between right‐sided and left‐sided RCC, in which a positive relationship exists between earlier stage and right‐sided disease. Therefore, right‐sided RCC has better CSS than left‐sided RCC, and laterality is also an independent prognostic factor for survival in the ≥10 cm subgroup. However, why a survival difference in disease laterality exists for RCC remains uncertain. Future research that can incorporate molecular and genetic elements of the disease will provide important insights into how tumor location is associated with the patient outcome.

Although the present study was large and investigated the different prognosticators of laterality and survival in RCC patients, several limitations exist. First, the study was retrospective study and lacked standardization for diagnostic procedures, therapy, and follow‐up. Second, some information was unavailable, such as smoking status, laboratory and metastatic patterns of adjuvant therapy and treatment of recurrent disease. Third, the follow‐up time was not sufficiently long because data from 2010 were collected soon after the 7th edition of the TNM system. Moreover, no molecular data were reported in these cohorts of patients who might partly explain the laterality difference. The major strength of this study included the large sample size and that we captured all cases of surgically treated RCC. To our knowledge, this investigation is the largest study that evaluated the CSS of laterality in RCC patients.

## 5 CONCLUSION

In summary, we found the association between tumor laterality and outcome of surgical RCC, in which right‐sided disease was superior to left‐sided disease in the tumor size ≥10 cm, age <65 years, male, Caucasian race, clear cell carcinoma type, and radical nephrectomy subgroups. Moreover, Laterality remained as an independent prognostic factor for cancer‐specific survival in subgroup of tumor size ≥10 cm RCC.

## CONFLICT OF INTEREST

The authors declare no conflict of interest.

## AUTHOR CONTRIBUTIONS

Junxing Chen and Chin‐Lee Wu had full access to all the data in the study and take responsibility for the integrity of the data and the accuracy of the data analysis. Study concept and design: Junxing Chen and Chin‐Lee Wu. Acquisition, analysis, or interpretation of data: All authors. Drafting of the manuscript: Shengjie Guo and Kai Yao. Critical revision of the manuscript for important intellectual content: All authors. Statistical analysis: Xiaobo He, Shulin Wu and Yunlin Ye. Study supervision: Junxing Chen and Chin‐Lee Wu.

## Data Availability

The data that support the findings of this study are available from the corresponding author upon reasonable request.
