# Supplementary material for: Prognostic significance of laterality in renal cell carcinoma: A population‐based study from the surveillance, epidemiology, and end results (SEER) database
Source: Cancer Med. 2019 Aug 12;8(12):5629–37. doi: 10.1002/cam4.2484 (PMC6745836; doi:10.1002/cam4.2484)
Supplement: Supplementary file 1 [file CAM4-8-5629-s001.docx]

**Stable 1** Multivariate analysis in different subgroups of patients with renal cell carcinoma in SEER between 2010 and 2014 for cancer specific survival**.**

| **Covariate** |  | **Multivariate analysis** |  |
| --- | --- | --- | --- |
|  | HR | 95%CI | P value |
| Age＜65 years group | | | |
| **Sex, No. (%)** |  |  |  |
| Male |  |  |  |
| Female | 0.97 | 0.86 to 1.10 | 0.671 |
| **Race, No. (%)** |  |  |  |
| American Indian/Alaska Native | ref |  |  |
| Asian or Pacific Islander | 1.95 | 1.01 to 3.77 | **0.046** |
| Black | 2.33 | 1.22 to 4.43 | **0.009** |
| White | 2.01 | 1.07 to 3.75 | **0.027** |
| Unknown | 0.42 | 0.05 to 3.28 | 0.408 |
| **AJCC Stage** |  |  |  |
| I | ref |  |  |
| II | 1.45 | 1.04 to 2.02 | **0.027** |
| III | 4.35 | 3.39 to 5.60 | **＜0.001** |
| IV | 21.2 | 16.5 to 27.3 | **＜0.001** |
| **Tumor size (cm)** |  |  |  |
| 0.1-3.9 | ref |  |  |
| 4.0-6.9 | 2.12 | 1.59 to 2.83 | **＜0.001** |
| 7.0-9.9 | 2.67 | 1.96 to 3.64 | **＜0.001** |
| ≥10 | 3.41 | 2.51 to 4.62 | **＜0.001** |
| **Histology** |  |  |  |
| Clear cell | ref |  |  |
| papillary | 1.38 | 1.10 to 1.74 | **0.005** |
| Collecting duct | 3.38 | 2.09 to 5.46 | **＜0.001** |
| Chromophobe | 0.47 | 0.29 to 0.76 | **0.002** |
| Other specified | 1.60 | 1.41 to 1.82 | **＜0.001** |
| **Grade** |  |  |  |
| 1 | ref |  |  |
| 2 | 1.32 | 0.82 to 2.13 | 0.241 |
| 3 | 3.27 | 2.05 to 5.22 | **＜0.001** |
| 4 | 5.52 | 3.44 to 8.84 | **＜0.001** |
| **Surgery type** |  |  |  |
| Partial Nephrectomy | ref |  |  |
| Radical Nephrectomy | 1.94 | 1.46 to 2.56 | **＜0.001** |
| **Laterality** |  |  |  |
| Left | ref |  |  |
| Right | 0.98 | 0.87 to 1.09 | 0.748 |
| Male group | | | |
| **Age, y** |  |  |  |
| Age＜65 | ref |  |  |
| Age ≥ 65 | 1.24 | 1.12 to 1.37 | **＜0.001** |
| **Race, No. (%)** |  |  |  |
| American Indian/Alaska Native | ref |  |  |
| Asian or Pacific Islander | 2.29 | 1.19 to 4.40 | **0.012** |
| Black | 2.13 | 1.12 to 4.05 | **0.020** |
| White | 2.00 | 1.07 to 3.72 | **0.029** |
| Unknown | 0.58 | 0.12 to 2.65 | 0.482 |
| **AJCC Stage** |  |  |  |
| I | ref |  |  |
| II | 1.24 | 0.94 to 1.64 | 0.114 |
| III | 3.43 | 2.82 to 4.18 | **＜0.001** |
| IV | 15.43 | 12.6 to 18.9 | **＜0.001** |
| **Tumor size (cm)** |  |  |  |
| 0.1-3.9 | ref |  |  |
| 4.0-6.9 | 1.79 | 1.43 to 2.23 | **＜0.001** |
| 7.0-9.9 | 2.38 | 1.87 to 3.04 | **＜0.001** |
| ≥10 | 3.01 | 2.36 to 3.82 | **＜0.001** |
| **Histology** |  |  |  |
| Clear cell | ref |  |  |
| papillary | 1.48 | 1.24 to 1.77 | **＜0.001** |
| Collecting duct | 3.11 | 2.00 to 4.83 | **＜0.001** |
| Chromophobe | 0.38 | 0.23 to 0.63 | **＜0.001** |
| Other specified | 1.59 | 1.41 to 1.78 | **＜0.001** |
| **Grade** |  |  |  |
| 1 | ref |  |  |
| 2 | 1.10 | 0.77 to 1.56 | 0.593 |
| 3 | 1.93 | 1.36 to 2.73 | **＜0.001** |
| 4 | 3.86 | 2.71 to 5.49 | **＜0.001** |
| **Surgery type** |  |  |  |
| Partial Nephrectomy | ref |  |  |
| Radical Nephrectomy | 1.83 | 1.47 to 2.27 | **＜0.001** |
| **Laterality** |  |  |  |
| Left | ref |  |  |
| Right | 0.94 | 0.85 to 1.04 | 0.275 |
| White group | | | |
| **Age, y** |  |  |  |
| Age＜65 | ref |  |  |
| Age ≥ 65 | 1.27 | 1.16 to 1.40 | **＜0.001** |
| **Sex, No. (%)** |  |  |  |
| Male | ref |  |  |
| Female | 1.02 | 0.93 to 1.13 | 0.570 |
| **AJCC Stage** |  |  |  |
| I | ref |  |  |
| II | 1.25 | 0.97 to 1.61 | 0.083 |
| III | 3.57 | 2.99 to 4.27 | **＜0.001** |
| IV | 16.3 | 13.6 to 19.6 | **＜0.001** |
| **Tumor size (cm)** |  |  |  |
| 0.1-3.9 | ref |  |  |
| 4.0-6.9 | 1.83 | 1.49 to 2.25 | **＜0.001** |
| 7.0-9.9 | 2.37 | 1.89 to 2.97 | **＜0.001** |
| ≥10 | 2.97 | 2.38 to 3.72 | **＜0.001** |
| **Histology** |  |  |  |
| Clear cell | ref |  |  |
| papillary | 1.61 | 1.35 to 1.91 | **＜0.001** |
| Collecting duct | 2.91 | 1.77 to 4.77 | **＜0.001** |
| Chromophobe | 0.41 | 0.27 to 0.62 | **＜0.001** |
| Other specified | 1.57 | 1.42 to 1.74 | **＜0.001** |
| **Grade** |  |  |  |
| 1 | ref |  |  |
| 2 | 1.20 | 0.88 to 1.65 | 0.242 |
| 3 | 2.30 | 1.68 to 3.14 | **＜0.001** |
| 4 | 4.25 | 3.10 to 5.83 | **＜0.001** |
| **Surgery type** |  |  |  |
| Partial Nephrectomy | ref |  |  |
| Radical Nephrectomy | 1.89 | 1.53 to 2.32 | **＜0.001** |
| **Laterality** |  |  |  |
| Left | ref |  |  |
| Right | 0.93 | 0.85 to 1.02 | 0.125 |
| Clear cell carcinoma group | | | |
| **Age, y** |  |  |  |
| Age＜65 | ref |  |  |
| Age ≥ 65 | 1.42 | 1.29 to 1.56 | **＜0.001** |
| **Sex, No. (%)** |  |  |  |
| Male | ref |  |  |
| Female | 1.02 | 0.92 to 1.13 | 0.618 |
| **Race, No. (%)** |  |  |  |
| American Indian/Alaska Native | ref |  |  |
| Asian or Pacific Islander | 1.59 | 0.80 to 3.17 | 0.180 |
| Black | 1.48 | 0.74 to 2.97 | 0.261 |
| White | 1.51 | 0.78 to 2.92 | 0.210 |
| Unknown | 0.21 | 0.02 to 1.70 | 0.145 |
| **AJCC Stage** |  |  |  |
| I | ref |  |  |
| II | 1.12 | 0.87 to 1.45 | 0.342 |
| III | 2.80 | 2.36 to 3.32 | **＜0.001** |
| IV | 12.60 | 10.6 to 15.1 | **＜0.001** |
| **Tumor size (cm)** |  |  |  |
| 0.1-3.9 | ref |  |  |
| 4.0-6.9 | 1.86 | 1.52 to 2.28 | **＜0.001** |
| 7.0-9.9 | 2.46 | 1.97 to 3.08 | **＜0.001** |
| ≥10 | 3.02 | 2.41 to 3.79 | **＜0.001** |
| **Grade** |  |  |  |
| 1 | ref |  |  |
| 2 | 1.27 | 0.92 to 1.74 | 0.133 |
| 3 | 2.30 | 1.68 to 3.15 | **＜0.001** |
| 4 | 5.01 | 3.64 to 6.91 | **＜0.001** |
| **Surgery type** |  |  |  |
| Partial Nephrectomy | ref |  |  |
| Radical Nephrectomy | 1.68 | 1.38 to 2.05 | **＜0.001** |
| **Laterality** |  |  |  |
| Left | ref |  |  |
| Right | 0.96 | 0.88 to 1.06 | 0.499 |
| Radical Nephrectomy group | | | |
| **Age, y** |  |  |  |
| Age＜65 | ref |  |  |
| Age ≥ 65 | 1.24 | 1.13 to 1.35 | **＜0.001** |
| **Sex, No. (%)** |  |  |  |
| Male | ref |  |  |
| Female | 1.02 | 0.93 to 1.12 | 0.536 |
| **Race, No. (%)** |  |  |  |
| American Indian/Alaska Native | ref |  |  |
| Asian or Pacific Islander | 1.81 | 1.04 to 3.14 | **0.034** |
| Black | 1.92 | 1.11 to 3.30 | **0.018** |
| White | 1.75 | 1.03 to 2.97 | **0.036** |
| Unknown | 0.54 | 0.15 to 1.88 | 0.333 |
| **AJCC Stage** |  |  |  |
| I | ref |  |  |
| II | 1.24 | 0.98 to 1.58 | 0.069 |
| III | 3.59 | 3.02 to 4.26 | **＜0.001** |
| IV | 15.5 | 12.9 to 18.4 | **＜0.001** |
| **Tumor size (cm)** |  |  |  |
| 0.1-3.9 | ref |  |  |
| 4.0-6.9 | 1.45 | 1.19 to 1.77 | **＜0.001** |
| 7.0-9.9 | 1.89 | 1.53 to 2.33 | **＜0.001** |
| ≥10 | 2.40 | 1.95 to 2.96 | **＜0.001** |
| **Histology** |  |  |  |
| Clear cell | ref |  |  |
| papillary | 1.49 | 1.26 to 1.75 | **＜0.001** |
| Collecting duct | 3.30 | 2.24 to 4.85 | **＜0.001** |
| Chromophobe | 0.48 | 0.33 to 0.69 | **＜0.001** |
| Other specified | 1.62 | 1.47 to 1.78 | **＜0.001** |
| **Grade** |  |  |  |
| 1 | ref |  |  |
| 2 | 1.43 | 1.01 to 2.02 | **0.038** |
| 3 | 2.76 | 1.97 to 3.88 | **＜0.001** |
| 4 | 5.14 | 3.65 to 7.24 | **＜0.001** |
| **Laterality** |  |  |  |
| Left | ref |  |  |
| Right | 0.96 | 0.88 to 1.04 | 0.364 |
