# Supplementary material for: Prognostic significance of laterality in renal cell carcinoma: A population‐based study from the surveillance, epidemiology, and end results (SEER) database
Source: Cancer Med. 2019 Aug 12;8(12):5629–37. doi: 10.1002/cam4.2484 (PMC6745836; doi:10.1002/cam4.2484)
Supplement: Supplementary file 2 [file CAM4-8-5629-s002.docx]

**Stable 2** Multivariate analysis in group of patients with renal cell carcinoma (tumor size＜4cm) in SEER between 2010 and 2014 for cancer specific survival**.**

.

| **Covariate** |  | **Multivariate analysis** |  |
| --- | --- | --- | --- |
|  | HR | 95%CI | P value |
| **Age, y** |  |  |  |
| Age＜65 (N=11326) |  |  |  |
| Age ≥ 65(N=6691) | 2.36 | 1.73 to 3.22 | **＜0.001** |
| **Sex, No. (%)** |  |  |  |
| Male (N=11008) |  |  |  |
| Female (N=7009) | 0.84 | 0.60 to 1.16 | 0.306 |
| **AJCC Stage** |  |  |  |
| I (N=16835) |  |  |  |
| III (N=1021) | 2.71 | 1.76 to 4.16 | **＜0.001** |
| IV (N=161) | 14.6 | 8.35 to 25.6 | **＜0.001** |
| **Histology** |  |  |  |
| Clear cell (N=11567) |  |  |  |
| Papillary (N=2950) | 1.81 | 1.22 to 2.68 | **0.002** |
| Collecting duct (N=16) | 3.48 | 0.82 to 14.8 | 0.090 |
| Chromophobe (N=776) | 0.48 | 0.15 to 1.55 | 0.225 |
| Other specified (N=2708) | 1.16 | 0.78 to 1.72 | 0.445 |
| **Grade** |  |  |  |
| 1 (N=2884) |  |  |  |
| 2 (N=10858) | 0.77 | 0.46 to 1.28 | 0.325 |
| 3 (N=3958) | 1.41 | 0.84 to 2.38 | 0.189 |
| 4 (N=317) | 4.51 | 2.48 to 8.20 | **＜0.001** |
| **Surgery type** |  |  |  |
| Partial Nephrectomy (N=11879) |  |  |  |
| Radical Nephrectomy (N=6138) | 3.59 | 2.50 to 5.15 | **＜0.001** |
| **Laterality** |  |  |  |
| Left (N=8672) |  |  |  |
| Right (N=9345) | 1.08 | 0.80 to 1.46 | 0.578 |
