# Supplementary material for: Prognostic significance of laterality in renal cell carcinoma: A population‐based study from the surveillance, epidemiology, and end results (SEER) database
Source: Cancer Med. 2019 Aug 12;8(12):5629–37. doi: 10.1002/cam4.2484 (PMC6745836; doi:10.1002/cam4.2484)
Supplement: Supplementary file 3 [file CAM4-8-5629-s003.docx]

**Stable 3** Multivariate analysis in group of patients with renal cell carcinoma (4cm ≤ tumor size＜7cm) in SEER between 2010 and 2014 for cancer specific survival**.**

| Covariate |  | Multivariate analysis |  |
| --- | --- | --- | --- |
|  | HR | 95%CI | P value |
| **Age, y** |  |  |  |
| Age＜65 (N=7233) | ref |  |  |
| Age ≥ 65(N=5924) | 1.41 | 1.19 to 1.67 | **＜0.001** |
| **Sex, No. (%)** |  |  |  |
| Male (N=8429) | ref |  |  |
| Female (N=4728) | 1.04 | 0.87 to 1.24 | 0.641 |
| **AJCC Stage** |  |  |  |
| I (N=10214) | ref |  |  |
| III (N=2431) | 2.71 | 1.76 to 4.16 | **＜0.001** |
| IV (N=512) | 14.6 | 8.35 to 25.6 | **＜0.001** |
| **Histology** |  |  |  |
| Clear cell (N=8811) | ref |  |  |
| Papillary (N=1676) | 1.38 | 1.05 to 1.82 | **0.021** |
| Collecting duct (N=23) | 2.05 | 1.04 to 4.05 | **0.037** |
| Chromophobe (N=598) | 0.41 | 0.19 to 0.88 | **0.022** |
| Other specified (N=2049) | 1.51 | 1.24 to 1.84 | **＜0.001** |
| **Grade** |  |  |  |
| 1 (N=1166) | ref |  |  |
| 3 (N=4190) | 2.69 | 2.61 to 3.35 | **＜0.001** |
| 4 (N=848) | 14.4 | 13.9 to 21.8 | **＜0.001** |
| **Surgery type** |  |  |  |
| Partial Nephrectomy (N=3781) | ref |  |  |
| Radical Nephrectomy (N=9376) | 1.66 | 1.27 to 2.16 | **＜0.001** |
| **Laterality** |  |  |  |
| Left (N=6604) | ref |  |  |
| Right (N=6553) | 1.13 | 0.95 to 1.34 | 0.142 |
