# Supplementary material for: Prognostic significance of laterality in renal cell carcinoma: A population‐based study from the surveillance, epidemiology, and end results (SEER) database
Source: Cancer Med. 2019 Aug 12;8(12):5629–37. doi: 10.1002/cam4.2484 (PMC6745836; doi:10.1002/cam4.2484)
Supplement: Supplementary file 4 [file CAM4-8-5629-s004.docx]

**Stable 4** Multivariate analysis in group of patients with renal cell carcinoma (7cm ≤ tumor size＜10cm) in SEER between 2010 and 2014 for cancer specific survival**.**

| **Covariate** |  | **Multivariate analysis** |  |
| --- | --- | --- | --- |
|  | HR | 95%CI | P value |
| **Age, y** |  |  |  |
| Age＜65 (N=3323) | ref |  |  |
| Age ≥ 65(N=2502) | 1.24 | 1.06 to 1.45 | **0.005** |
| **Sex, No. (%)** |  |  |  |
| Male (N=3963) | ref |  |  |
| Female (N=1862) | 1.01 | 0.85 to 1.20 | 0.880 |
| **AJCC Stage** |  |  |  |
| I (N=527) | ref |  |  |
| II (N=2498) | 0.87 | 0.53 to 1.44 | 0.604 |
| III (N=1991) | 2.40 | 1.49 to 3.86 | **＜0.001** |
| IV (N=809) | 11.82 | 7.37 to 18.97 | **＜0.001** |
| **Histology** |  |  |  |
| Clear cell (N=3988) | ref |  |  |
| Papillary (N=505) | 1.17 | 0.83 to 1.65 | 0.361 |
| Collecting duct (N=21) | 5.51 | 2.92 to 10.4 | **＜0.001** |
| Chromophobe (N=259) | 0.29 | 0.12 to 0.70 | **0.006** |
| Other specified (N=1052) | 1.58 | 1.33 to 1.88 | **＜0.001** |
| **Grade** |  |  |  |
| 1 (N=315) | ref |  |  |
| 2 (N=2291) | 0.96 | 0.54 to 1.71 | 0.907 |
| 3 (N=2325) | 1.72 | 0.98 to 3.02 | 0.057 |
| 4 (N=894) | 3.68 | 2.09 to 6.50 | **＜0.001** |
| **Surgery type** |  |  |  |
| Partial Nephrectomy (N=456) | ref |  |  |
| Radical Nephrectomy (N=5369) | 1.03 | 0.68 to 1.56 | 0.880 |
| **Laterality** |  |  |  |
| Left (N=2933) | ref |  |  |
| Right (N=2892) | 0.96 | 0.83 to 1.13 | 0.689 |
